# The morphology of an intercalated Au layer with its effect on the Dirac point of graphene

**DOI:** 10.1038/s41598-020-57982-z

**Published:** 2020-01-23

**Authors:** Amirhossein Bayani, Karin Larsson

**Affiliations:** Department of Chemistry-Ångström laboratory, Uppsala University, Uppsala, Sweden

**Keywords:** Physical chemistry, Theoretical chemistry, Electronic and spintronic devices, Graphene, Atomistic models

## Abstract

This is a theoretical investigation where Density Functional Theory (DFT) has been used in studying the phenomenon of Au intercalation within the 4H-SiC/graphene interface. The electronic structure of some carefully chosen morphologies of the Au layer has then been of special interest to study. One of these specific Au morphologies is of a more hypothetical nature, whilst the others are, from an experimental point of view, realistic ones. The latter ones were also found to be energetically stable. Band structure calculations showed that intercalated Au layers with morphologies different from a planar Au layer will induce a band gap at the Dirac point of graphene (with up to 174 meV for the morphologies studied in the present work). It should here be mentioned that this bandgap size is four times larger than the energy of thermal motion at room temperature (26 meV). These findings reveal that a wide bandgap at the Dirac point of graphene comes from an inhomogeneous staggered potential on the Au layer, which non-uniformly breaks the sublattice symmetry. The presence of spin-orbit (SO) interactions have also been included in the present study, with the purpose to find out if SO will create a bandgap and/or band splitting of graphene.

## Introduction

Intercalation processes have recently made it possible to produce large quasi free-standing graphene layers on different substrates^1–5^. One method, which is based on thermal annealing, uses a 4H-SiC (0001) substrate with an attached carbon buffer layer onto the Si-face of the substrate. Various types of metals have then been used with the purpose to intercalate these metal atoms between SiC and the buffer layers, thereby creating a monolayer (ML) of graphene with an intact Dirac point (DP)^6^. The few existing Si-C covalent bonds between the 4H-SiC (0001) substrate and the carbon buffer layer will then break (i.e., approximately 20% of the buffer layer C atoms are involved in covalent bonds with the Si atoms on the SiC substrate surface). In relation to the majority of the buffer layer C atoms that are sp^2^-hybridized, those C atoms that bind to the SiC substrate are sp^3^-hybridized. This type of partial sp^3^-hybridization (i.e., involving just a smaller part of the buffer layer C atoms) makes graphene act as an insulator without any Dirac cone^7^. Moreover, when positioning heavy metal atoms under graphene, the spin-orbit coupling will increase that causes band splitting^8^ (e.g., of approximately 70 meV with an intercalated Au layer. This phenomenon comes from the breakage of a mirror symmetry due to interaction with the substrate^9–12^ (which increases the Rashba splitting in graphene). A giant Rashba splitting is a very important property for a material to be used in the design of spintronic devices^13,14^. Perturbation theory has also earlier been used with the purpose to show that electrons within a p_z_ orbital of graphene can be transferred to another p_z_ orbital of graphene, either in pristine graphene or by using one of the d-orbitals, in the intercalated gold layer, as a bridge^9^.

In several earlier investigations, one has been trying to create a bandgap at the Dirac point of graphene by applying strain, spatial restriction, or by breaking the sublattice symmetry^15–20^. Only a few of these studies resulted in the opening of a wide and controllable bandgap that is larger than the energy of thermal motion at room temperate. To the knowledge of the authors, the role of the morphology of the intercalated layer, on the Dirac point of graphene, has not yet been considered.

The main goal of the present study has been to show how the morphology of the intercalated Au layer will affect the electronic properties of a monolayer (ML) of graphene. The possibility to induce a band gap at the Dirac point of graphene, by manipulating the staggered potential of a 4H-SiC/Au substrate, ha thereby been looked for. For this purpose, (i) several tilted Au layers, (ii) a zig-zag-shaped Au layer, and (iii) a mixed Au-Ag layer were used. These layers are monolayers, except (ii). This is a novel idea, which shows that the morphology of an intercalated layer can change the size of the bandgap at the Dirac point of graphene. The calculations were based on a highly accurate density functional theory (DFT) method, by which both the geometrical and electronic properties of the structures were obtained.

## Results and Discussion

### Structure information

**Figure 1:**
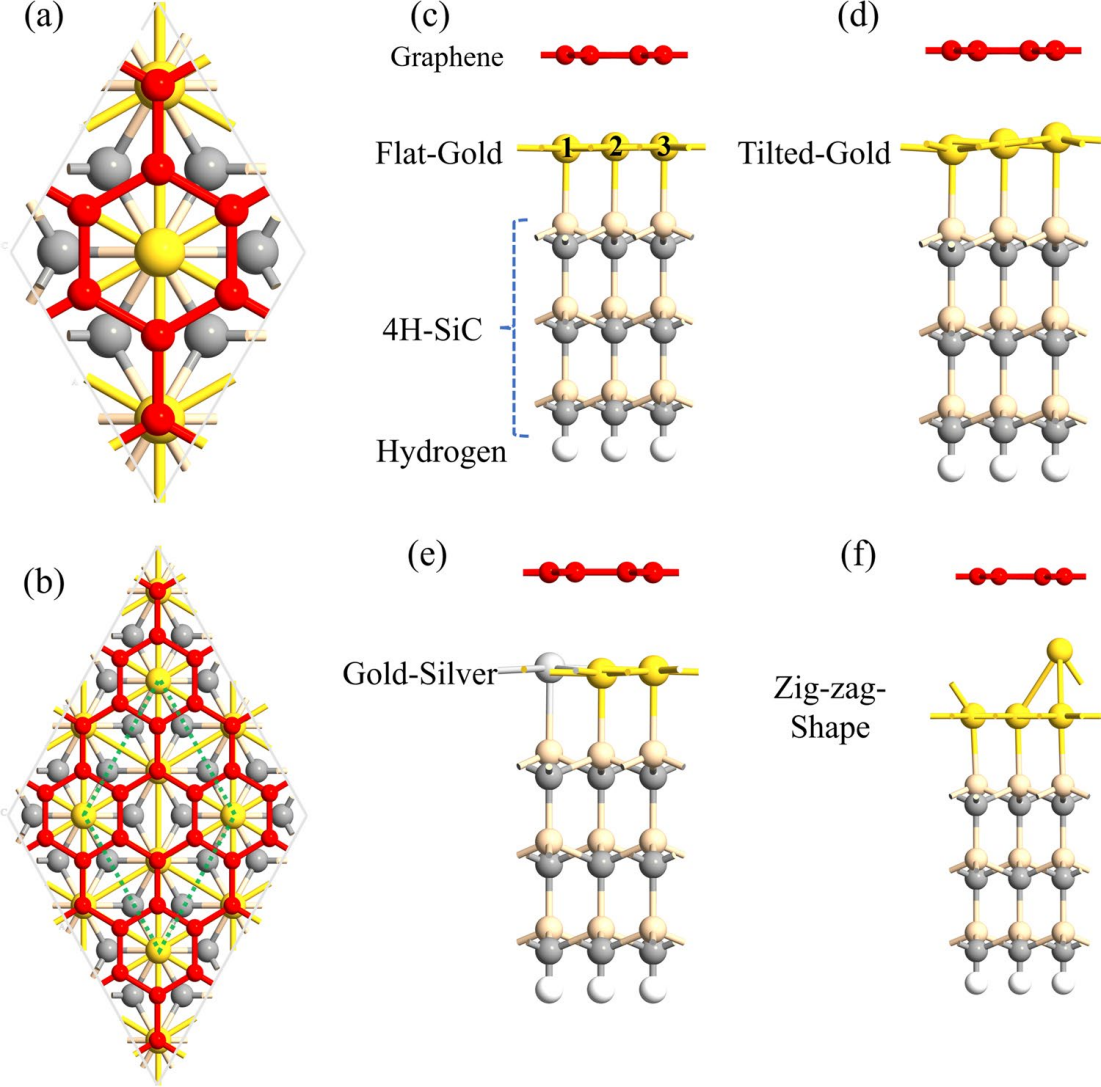
(**a**,**b**) Show the top views of the SiC/Au/graphene configuration (with an Au coverage of 1/3 ML) of different unit cell sizes. (**c**–**f**) Present the side views of the structures, corresponding to a flat gold layer, a tilted gold layer, a mixed gold-silver layer, and a zig-zag-shaped gold layer, respectively. Red: graphene; Yellow: gold; Light yellow: silicon; Gray: carbon; White: hydrogen; Light grey; silver.

The supercell models in the present study were based on monolayers of Au that were initially; i) flat, ii) slightly tilted, iii) built as a monoatomic step, or iv) intermixed with Ag within the monolayer (Fig. 1). Model types (ii) and (iii) are globally observed as zig-zag formations to various extents. As a first step in the present investigation, the relaxed structure of 4H-SiC/Au/graphene, with a flat Au monolayer, was studied. As can be seen in Fig. 1a, the graphene overlayer was also rotated with respect to SiC, forming a unit cell of $\sqrt{3}\times \sqrt{3}R{30}^{^\circ }$. In this structure, there are three gold atoms positioned under eight carbon atoms of graphene, creating a 1/3 ML of Au atoms (with a side view presented in Fig. 1c). The calculated vertical distance between the gold layer and graphene was found to be approximately 2.87 Å, which is different from the value obtained in refs. ^21–23^. (with distances larger than 3 Å). The theoretical work performed in these references were based on the same model as in the present investigation. However, they used the Projector Augmented Wave method (DFT-PAW) and the VASP software. This is different from the software and method that have been used in the present study (Atomistix ToolKit 2018.1 with the linear combination of atomic orbitals method (DFT-LCAO)). Due to this difference in results, another theoretical parameter was tested (i.e., using the RPBE instead of the PBE functional, and using DFT-D3 instead of DFT-D2). It must here be emphasized that the usage of different methods did not alter the Au/graphene distance to any important extent. The distance was still below 3 Å (i.e., 2.92 Å).

### Initially planar Au layer

**Figure 2:**
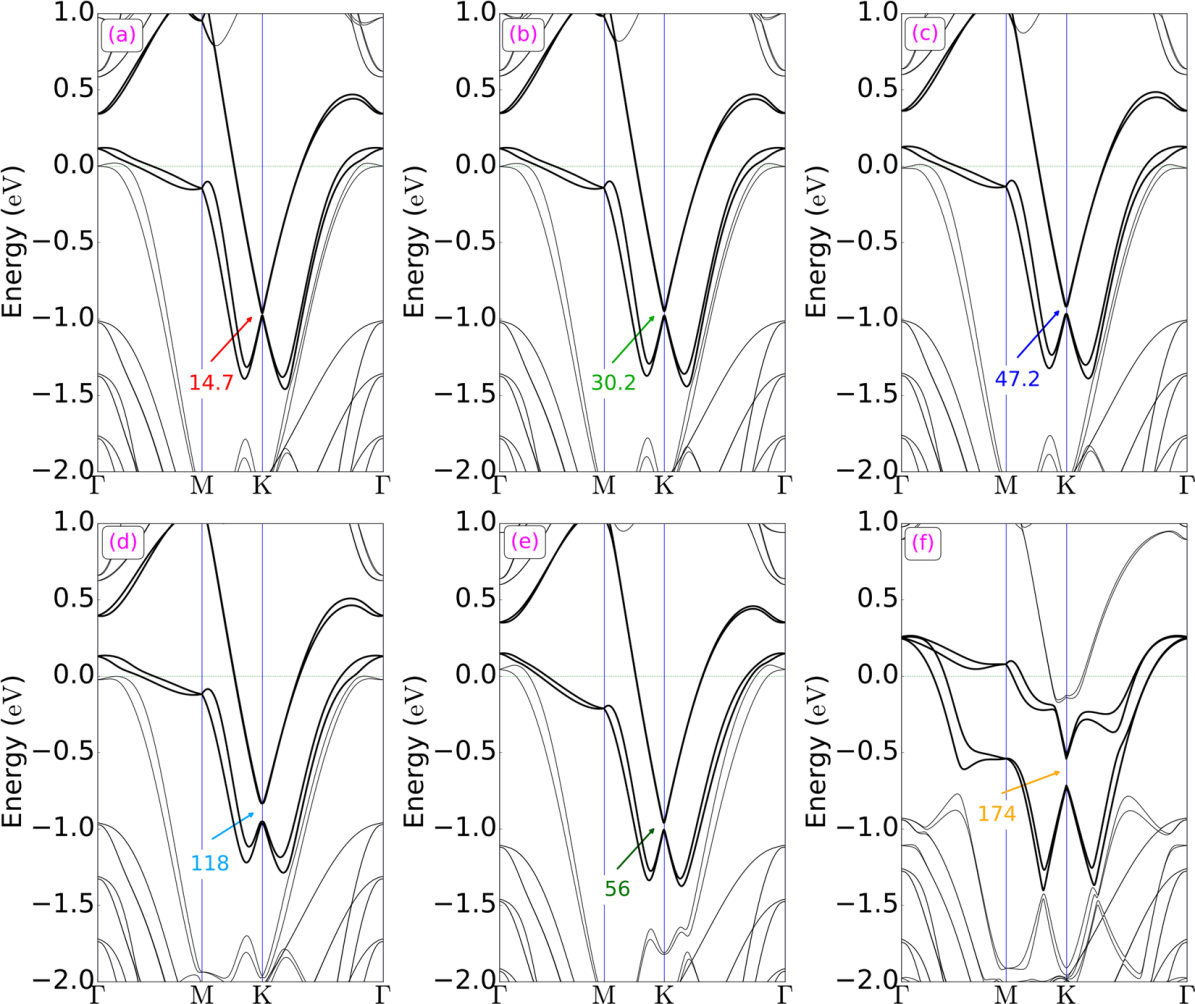
Band structures of SiC/Au/graphene for a flat Au (**a**), tilted Au (**b**–**d**), mixed Au-Ag (**e**), and zig-zag-shaped Au (**f**) layers in the Γ-M-K-Γ direction. The bandgap opening at the K point (induced by breaking the sublattice symmetry in graphene) and a band splitting (due to spin-orbit interactions) are here demonstrated. The bands that are related to the Dirac point of graphene are shown in bold, and the numerical values of the bandgap sizes (in meV) are marked in the respective figure.

**Figure 3:**
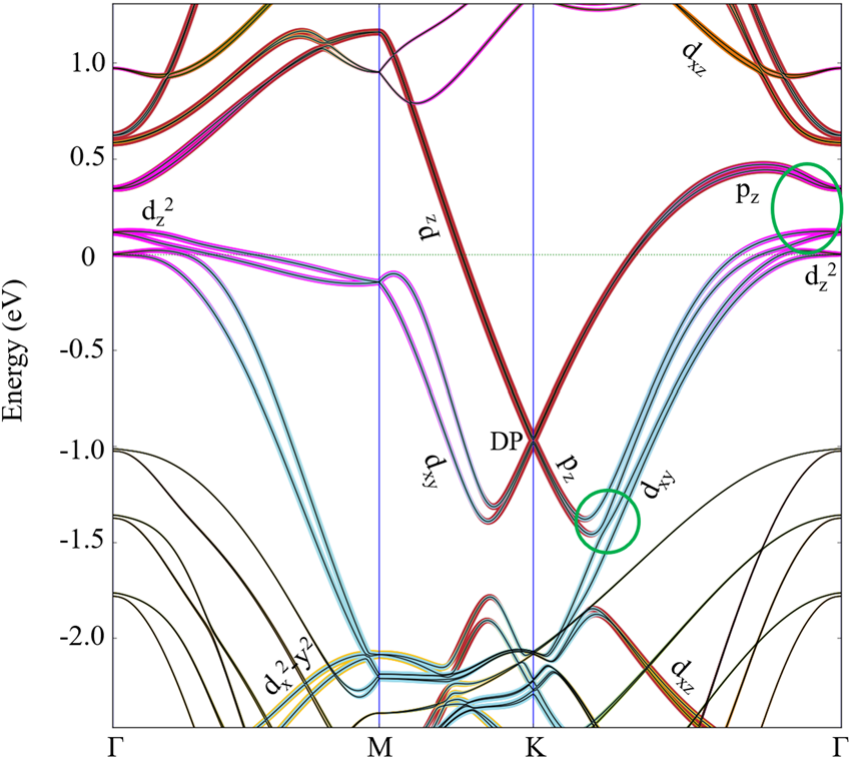
Analysis of projected band structures (including spin-orbit interactions) for SiC/Au/graphene with a flat Au layer (as shown in Fig. 1c). As a reference, the black lines represent the total band structure of 4H-SiC (0001)/Au/graphene. The Dirac point (DP) of graphene is positioned 0.96 eV under the Fermi level energy (which is set to zero). Red: p_z_ orbitals; Purple: d_z_^2^ orbitals; Cyan: d_xy_ orbitals; Yellow: d_x_^2^-y^2^ orbitals; Orange: d_xz_ orbitals. The green circles represent the hybridization between the p_z_ orbital and the d_z_^2^ and d_xy_ orbitals.

The band structure was calculated for the relaxed 4H-SiC/Au/graphene model, with a planar Au monolayer, including also spin-orbital interactions (see Fig. 2a). It can in this Figure be seen that the Dirac point (DP) of graphene is located at −0.96 eV (the experimental value is −0.85 eV^4^). This result is an indication of n-type doping, which has also earlier been observed for graphene^21^ (the nature of doping will be further explained in Section *Zig-zag-sized Au layer*). The difference between the here calculated position of DP and the experimental results is originating from different gold-graphene distances. As can also be seen in Fig. 2a, an induced bandgap at the DP of graphene is 14.7 meV, in addition to a band splitting of 10 meV. This band splitting is due to the hybridization between the p_z_ orbitals of the C atoms (in graphene) and two types of 5d-orbitals (d_z_^2^ and d_xy_) from the Au atoms (in the gold layer). In order to visualize the hybridization of these orbitals, the projected band structure analysis was performed. The band structure was thereby projected onto the p_z_ orbitals of the graphene carbon atoms, and onto the 5d-orbitals of the Au atoms. As can be seen in Fig. 3, the p_z_ orbitals of the C atoms hybridize very efficiently with the d_z_^2^ and d_xy_ orbitals of Au. At the Γ point, the hybridization between p_z_ and d_z_^2^ is stronger than the hybridization between p_z_ and d_xy_, whereas the hybridization between p_z_ and d_xy_ is more substantial closer to the K point. Hybridization schemes involving the other Au d-orbitals (i.e., d_xz_ and d_yz_) were observed to occur far from the DP (i.e., far from E = −0.96 eV).

**Figure 4:**
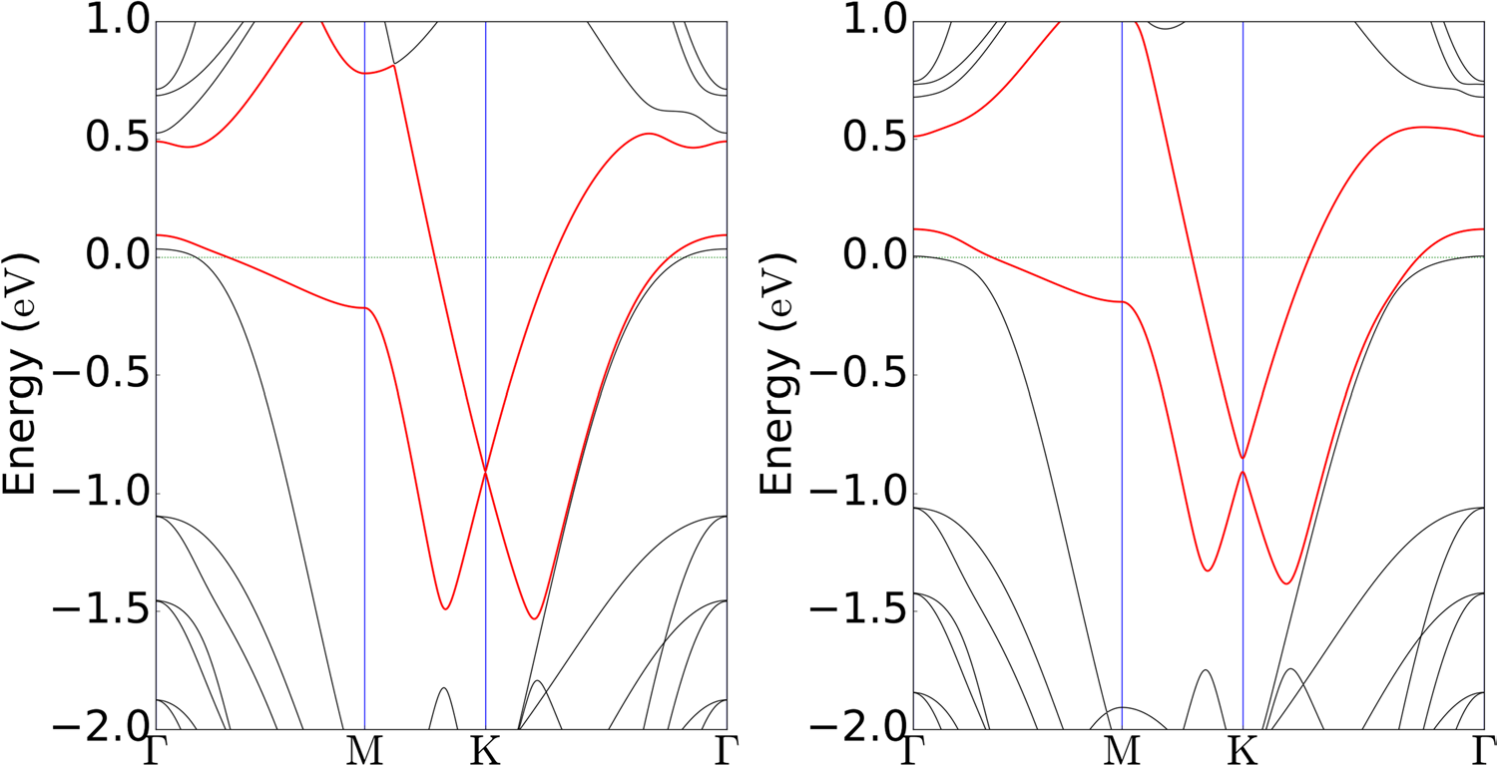
Band structures (without spin-orbit interactions) of flat (left) and tilted Au (right) layers in a SiC/Au/graphene structure (as shown in Fig. 2a,d). Variations in morphology of the intercalated Au layer are here shown to cause a bandgap opening at the K point for graphene. Red lines indicate the p_z_ orbital of the graphene carbon atoms.

It was at this stage very interesting to outline the underlying causes of the bandgap opening at the DP of graphene. To achieve a deeper understanding of this phenomenon, the band structure calculations had to be remade, but without any spin-orbit interactions. As a result, the value of the bandgap did not noticeably change; from 14.7 meV (with spin-orbit interactions) to 13.9 meV (without spin-orbit interactions) (see Fig. 4). And as expected, the band splitting disappeared due to the ignorance of spin degeneracy^24^. Hence, it can be concluded that spin-orbit coupling will not result in a bandgap opening in graphene.

### Role of Au morphology

The next step in the present study was to investigate the possibility that differences in the Au atoms position (i.e., the morphology of the Au layer) might be the cause of the observed bandgap opening. The background to this hypothesis was, that a closer analysis of the geometrical structure of the initially flat Au monolayer revealed that minor changes in the relative z-positions had occurred as a result of the geometry optimization of the 4H-SiC/Au/graphene model. More precisely, the positions of Au2 and Au3, as indicated by 2 and 3 in Fig. 1c, became identical in the z-direction. However, the z-position of Au1, as indicated by 1, was somewhat lower (with a difference of 0.058 Ǻ). Hence, as a result of the geometry optimization of an intercalated planar Au monolayer, a somewhat rougher and non-planar Au plane was formed. The suggestion was now that this structural disrupture, from a perfectly flat Au layer, will most probably cause the observed bandgap opening at the DP of graphene. To test this hypothesis, variously tilted gold layers were considered (Fig. 1d). More precisely, these tilted Au layers were constructed in the supercell, thereby forming zigzag-patterned Au surfaces with various degrees of roughness. The purpose of these more artificial surface structures was solely to outline the effect of the intercalated Au layer morphology on the electronic properties of graphene.

The band structures of these artificial structures were accordingly calculated, and the results confirmed that by increasing the tilting angle in the Au layer (i.e., by changing the roughness of the intercalated Au layer), the size of the bandgap at the Dirac point of graphene will increase to a quite large extent. Even tilting at a smaller degree, with a Δz value of 0.35 Å for Au3, was found to create a wide bandgap of size 118 meV (as shown in Fig. 2d). Similar to the flat Au layer, spin-orbit coupling did not have any significant role in creating the bandgap (see Fig. 4). To make sure that the unit cell size did not affect the results, larger unit cells of both flat and deliberately tilted gold layers were also considered. Results from these calculations, without considering spin-orbit coupling, can be seen in Fig. 1b. A projected band structure analysis confirmed that a staggered potential is the only underlying cause of the bandgap opening and that changing the unit cell size will not have any effect on the bandgap size (see Fig. S1 provided in the Supporting Information).

A look at the Tight Binding (TB) Hamiltonian of graphene can be very helpful in describing the bandgap opening. Here, Eq. 1 shows Hamiltonian without spin-orbit interactions^25^.

1 $${H}_{D}={H}_{0}+{H}_{SP}$$

where *H*_*D*_, *H*_0_ and *H*_*SP*_ describe the orbital dispersion, the effective Hamiltonian, and the staggered potential Hamiltonian, respectively^25^. *H*_0_ gives a gapless Dirac state with a conical dispersion ${\varepsilon }_{0}=\alpha h{v}_{f}|k|$ at the Dirac point, and *H*_*SP*_ is related to the effective orbital energy difference between the A and B graphene sublattices. The latter Hamiltonian causes an energy gap (i.e., bandgap) at the Dirac point of 2ξ (ξ is a parameter which controls the proximity-induced energy gap). Moreover, the orbital dispersion, *H*_*D*_, is equal to ${\varepsilon }_{D}=\alpha \sqrt{{\xi }^{2}+{(\alpha h{v}_{f}|k|)}^{2}}$ [α = 1 (or −1) for the conduction (or valence) band]. As was mentioned in refs. ^14,26^, the term for the staggered potential, *H*_*SP*_, can break the A and B sublattice symmetry in graphene, and thereby create a bandgap at the DP of graphene. This is due to a breakage of the space inversion symmetry, and a chemical equivalence to these A and B lattice sites^27^ are observed for a graphene monolayer adhered to an h-BN (0001) sheet^28^. Hence, a monolayer of graphene being adhered to an Au monolayer, which is the situation in the present investigation, will ultimately play a very important role in the calculated results in the present study. However, another factor (besides this type of staggered potential) will also affect the bandgap size at the Dirac point of graphene, and this factor is the inhomogeneity in the gold-graphene interactions within the x-y plane. As an explanation of what this means for the present model systems, by altering the degree of morphology (i.e., roughness) of the intercalated Au layer, the staggered potential (*H*_*SP*_) will also be altered, and in an identical manner. In short, by breaking the sublattice symmetry in graphene in an asymmetry way, a sizable band gap at the Dirac point of graphene will be formed. If we compare Fig. 1c,d, there is a staggered potential due to gold layers in both structures. However, in Fig. 1d the staggered potential is more non-uniform, which is the main cause of the larger bandgap at the DP.

### A mixture of Au and Ag

To further illuminate the importance of an inhomogeneous staggered potential in creating the bandgap, a flat atomic layer that consists of both Au and Ag atoms has been considered (Fig. 1e). The band structure of this system reveals that although a flat layer was considered, a gap of 56 meV was formed at the DP of graphene. This is due to the different potentials created by the different atoms Au and Ag (see Fig. 2e). The usage of an inhomogeneous staggered potential leads to an increased ξ value (Eq. 1), which in turn opens up a wide bandgap.

### Au/graphene distance effect

Since the distance between the gold layer and graphene was, in several experimental studies, reported as larger than 3 Å, the band structure of the 4H-SiC/Au/graphene model, given in Fig. 2b, has here been re-calculated with an Au-graphene distance of 3.0 and 3.2 Å, respectively. The geometry of the systems was then optimized, with a fixed Au and graphene distance. The resulting band structures did again confirm that different roughness of the intercalated Au monolayer will cause a bandgap opening at the Dirac point of graphene (as can be seen in the Supporting Information; Fig. S2). The main difference in results was that the size of the bandgap opening will decrease with an increase in Au-graphene distance. This is an expected result since the interactions between the gold layer and graphene will decrease with a longer gold-graphene distance. In addition, the position of the Dirac point of graphene changed by increasing the Au-graphene distance. The Dirac point was positioned at −0.82 eV (i.e., below the Fermi energy) for the distance of 3.2 Å, which agrees with the experimental value of −0.85 eV^4^. Due to the strong effect of the Au-graphene distance on the bandgap opening, the given band structures in Fig. 2 have been re-calculated for the larger Au-graphene distance of 3.2 Å. Figure S3 (provided in the supporting information file) clearly shows that the gap opening at the Dirac point strongly depends on the Au-graphene distance.

### Zig-zag-sized Au layer

After these observations, it was quite natural to continue the investigation by also introducing a more realistic model of an asymmetric 4H-SiC/Au/graphene structure (i.e., experimentally feasible). Within the supercell, an Au layer with a monoatomic step was thereby formed. The geometry optimized structure thereof can be seen in Fig. 1f. The drawback of these types of careful DFT calculations is the limitation in supercell model sizes. As a consequence, the model, seen from a more global perspective, will be a surface with a very large number of monoatomic steps that look like a zig-zag pattern. During experimental conditions, these types of monoatomic steps will be formed, but to a smaller extent. In addition, there will also be other forms of surface defect sites and asymmetric morphologies that will cause a non-uniform staggered potential. However, the purpose of the present study has been to solely demonstrate how asymmetry in the surface morphology might affect the possibility for a bandgap opening at the DP of graphene, and these surface steps are plausible examples of these types of asymmetries. Understandingly, it is not possible to more accurately model the very complex nature of a 4H-SiC/Au/graphene structure with unspecified surface morphology. A very well-controlled growth of the 4H-SiC/Au/graphene system, as well as a very careful characterization thereof, are needed for a more detailed and accurate theoretical prediction to take place.

The calculated band structure for this 4H-SiC/Au/graphene structure, with monoatomic thick steps on the intercalated Au layer, showed similar results as for the other Au morphologies, but to a larger extent. The resulting size of the bandgap at the DP of graphene was 174 meV (see Fig. 2f), which is six times larger than the energy of thermal motion at room temperature (26 meV). It is tempting to draw some experimental-related conclusions from these results. For instance, this value brings a low off-current for electronic devices that are based on graphene. The advantage of this model is that the Dirac cone is kept intact, which is extremely important for the graphene-based applications in electronics.

To verify the dynamic stability of the SiC/Au/graphene system, with a monoatomic step on the intercalated Au layer, the phonon band structure was also calculated. As can be seen in Fig. S4 in the Supporting Information, all phonon branches were found to be positive, with no appearance of imaginary phonon modes. The absence of negative bands proves the stability of the proposed SiC/Au/graphene structure. The nature of n-doping was also investigated for the SiC/Au/graphene system (with a monoatomic step on the intercalated Au layer), using the Mulliken Population analysis. It shows that the gold layer has donated electrons to the graphene layer (partially) to an extent of 0.23 e, and the main part comes from the d_z_^2^ orbitals on the Au atoms.

The values of the bandgap and band splitting, as well as the position of the Dirac point for each considered 4H-SiC/Au/graphene structure, are summarized in Table 1. As can be seen in this Table, the most striking result is that a zig-zag-shaped intercalated Au layer will increase the bandgap and band splitting (of graphene) simultaneously, as compared with a flat layer of Au. These are properties which are highly desirable for both electronic and spintronic devices^29^.

**Table 1:** The tilting size (Δ) (in Å), Dirac point position (D_p_) (in eV), as well as values of bandgap (E_g_) and band splitting (B_sp_) at the Dirac point of graphene (in meV), are here presented for each 4H-SiC/Au/graphene system (as shown in Fig. 2).

Configuration	Δ	E_g_	B_sp_	D_p_
Flat-gold	—	14.7	10.0	−0.96
Tilted-gold	0.11	30.2	8.0	−0.95
Tilted-gold	0.17	47.2	7.7	−0.94
Tilted-gold	0.35	118.0	4.8	−0.88
Gold-silver	—	56.0	5.5	−0.97
Zig-zag-gold	—	174.0	22.8	−0.61

The tilting size is here defined as the difference between the Au atoms positions 1 and 3 (see Fig. 1c).

**Figure 5:**
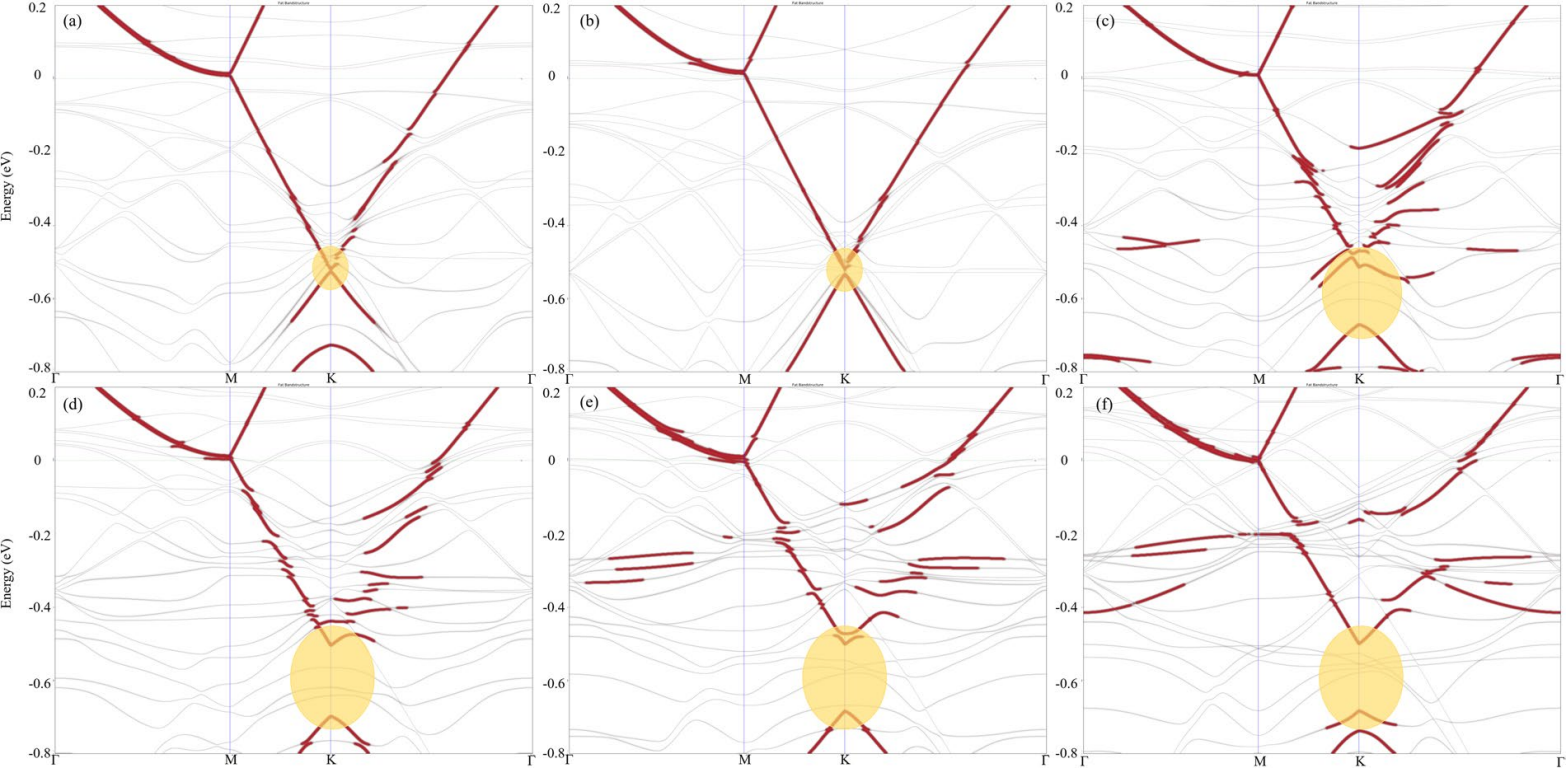
A projected band structure analysis shows here how the bandgap at the Dirac point of graphene will change when increasing the inhomogeneity length (IL). The bandgap will at first increase appreciably (when increasing IL from 0.29 nm (**a**) to 1,64 nm (**d**)). It will thereafter slightly decrease until an IL of 2.53 nm is reached (**f**). (**b**–**e**) correspond to an IL of 0.75, 1.19, 1.64, and 2.9 nm, respectively. Red lines: p_z_ orbitals of graphene; black lines: total bandstructure; yellow areas: bandgap opening at the Dirac point of graphene.

Moreover, it is also interesting to study the effect of inhomogeneity length on the bandgap opening at the DP of graphene. Additional calculations were thereby made, using a larger supercell of SiC/Au/graphene (with a $4\sqrt{3}\times 4\sqrt{3}R{30}^{^\circ }$ supercell matching of graphene to SiC) (see Fig S5a in the Supporting Information). This new supercell was used as the main structure in the construction of periodic 2D models with varying concentrations of island formations (out of which each included mono-atomic steps). Figure S5a was thereby successively filled (from the bottom) from 0.29 nm to 2.53 nm, and the so-called inhomogeneity length (IL) was thereby formed (the full size of the “homogeneity” is here 3.28 nm in the y-direction). The band structures for the inhomogeneity lengths of 0.29, 0.75, 1.19, 1.64, 2.09, and 2.53 nm were thereafter calculated, and the corresponding bandgap openings (at the DP of graphene) were obtained. As can be seen in Fig. 5, by increasing IL from 0.29 nm to 1.64 nm, the size of the bandgap at the DP of graphene was found to drastically increase; from 5.7 meV to 196.1 meV. Additional increments in IL were observed to only slightly decrease the bandgap, which is shown in the Supporting Information (see Fig. S5b). The most interesting result here is that the largest band gap will be obtained when half of the Au sheet is covered with two-layer formations of triangular islands. Also, due to the strong effect of the Au-graphene distance on the size of the energy gap at the Dirac point of graphene, the bandstructure for IL=1.64 nm has been re-calculated for an Au-graphene distance of 3.2 Å. The results showed again a decrease in the size of the bandgap, and it now became 142.6 meV.

It has earlier been reported that when experimentally depositing a monolayer of graphene onto a step-layer of Ir (111), the graphene layer was observed to follow the substrate shape. Hence, it was bent at the step edges on the Ir substrate, maintaining a constant Ir-graphene distance^30^. However, this type of graphene curvature (or bending) of the graphene monolayer was not observed in the present study. There are two plausible explanations for this circumstance. One of these relates to the very strong intra-bonds (i.e., covalent bonds, with delocalized p_z_ electrons) within graphene, which is to be compared with the much weaker Au-graphene bonds. The other explanation is the very large concentration of step-edges on the Au substrate. There is, thus, not enough length (along the x-axis) of the lower Au surface, for it, with its weak attraction force towards the graphene monolayer, to pull the graphene sheet closer to itself. The maximum lower-surface length in the present study was 3.28 nm, while it was 60 nm in the Ir example^30^. Simulation of such a large model is, though, practically impossible when using DFT techniques.

The here presented results clearly show that it is possible to control the width of the bandgap by controlling the surface morphology of the intercalated gold layer. Even though a truly experimental 4H-SiC/Au/graphene system has not been modeled, with all of its plausible defects and asymmetric morphologies, it is possible to draw some major conclusions from the present results;

- By depositing more than one intercalated gold layers, it will be possible to control the bandgap at the Dirac point of graphene.
- By increasing the thickness of the gold layer steps, it will both be possible to control the bandgap at the DP of graphene, as well as shifting the DP to a positive value. This brings p-type doping in graphene^21,31^. (Within the present study, the usage of the zig-zag-layered Au changed the position of the Dirac point from 0 to −0.61 eV).

## Conclusions

The effect by the morphology of an intercalated Au layer on the bandgap at the Dirac point of graphene has here been investigated for the 4H-SiC/Au/graphene system by using first principle DFT calculations. The gold layer was thereby used as an intercalated layer within the 4H-SiC (0001)/graphene interface. It was shown that wide bandgaps can be created by the formation of different types of Au morphologies of nanosized dimensions. The Dirac cone near the K point of graphene was, however, observed to be intact, and did thus not vary with the different nanostructures of the gold layer. The inhomogeneous staggered potentials that were created by either a nano-structured Au intercalating layer or by a mixed Ag-Au intercalating monolayer, confirmed that a uniformly staggered potential at the 4H-SiC/Au surface cannot induce a wide gap at the Dirac point of graphene. Instead, the breakage of the graphene sublattice symmetry to an asymmetric form of the 4H-SiC (0001)/Au substrate potential, is the key parameter for creating this type of bandgap.

Within the present study, the most important nanostructured Au layer was constructed with monoatomic thick steps (in the supercell) on the surface. It is therefore regarded to be an experimentally feasible surface, and calculations of the phonon band structure verified the stability of this specific SiC/Au/graphene structure. This type of zig-zag-formation (globally seen) resulted in an inhomogeneous staggered potential, that in turn acted upon the adhered graphene monolayer and caused the bandgap opening. In addition to these observations, spin-orbit interactions also resulted in a band splitting at the Dirac point of graphene. The hybridizations between the p_z_ orbitals on the C atoms (in graphene) with the d_z_^2^ and d_xy_ orbitals on the Au atoms (in the gold layer), are the main causes to this type of effect. However, the spin-orbit interactions were not observed to have any important effect on the size of the bandgap at the Dirac point of graphene. We also found a strong connection between the size of the bandgap at the Dirac point of graphene and the distance between the gold layer and the graphene sheet. The size of the gap became reduced by increasing the Au-graphene distance. The here presented results can be useful, not only in diverse scientific fields (like electronics, spintronics, sensors, etc.) but also within future electronics industries that are based on 2D materials.

## Methods and Models

The DFT calculations were based on a generalized gradient approximation (GGA)^32,33^, using a pseudopotential in addition to a linear combination of atomic orbitals (LCAO). (The software Atomistix ToolKit 2018.1 was used for these calculations^34^). The pseudopotential used in the calculations was the norm-conserving pseudopotential (SG15) which includes multiple projectors, semi-core states, and a non-linear core correction with fully relativistic features^35,36^. Also, to describe the Van der Waals forces between graphene and the gold layer, DFT-D2 was added to the calculations^37^. Moreover, a dense Monkhorst-Pack k-point sampling (35 × 35 × 1) was used in calculating the band structure and the projected band structure (as proposed in ref. ^38^). This number of k-points was here regarded to be accurate enough in the description of the present types of model systems, including both d-orbitals within Au, as well as the Dirac point of graphene^28,38^. To avoid malicious effects (such as eggbox effects^39^), the density mech cut-off was set to a quite large value; 300 Ha (8163 eV)^40^. Since the spin-orbit interaction is an intrinsic effect, the Fermi-Dirac occupation method, with a broadening of 50 K (0.0043 eV), was used in describing the electronic properties of the system with high precision. To evaluate the choice of basis set, the band structures were calculated by using also a larger basis set (i.e., with more shells and atomic orbitals) in the DFT calculations; SG15-High-BasisSet. No significant changes were thereby observed, both with and without the inclusion of spin-orbit interactions.

When constructing the unit cell model of the 4H-SiC (0001)/Au/graphene interface, the method proposed in ref.^21^ was used. To match the graphene monolayer to the underneath positioned SiC/Au structure, a tensile strain of 8.4% was considered (i.e., due to the larger unit cell of SiC/Au as compared to graphene). This value is in agreement with previous theoretical investigations^21,41^. In this matching procedure, a (2 × 2) unit cell of graphene was adjusted to the unit cell of 4H-SiC (0001) (which is shown in Fig. 1). This resulted in a slab configuration, where a vacuum layer of size 60 Å was used (in the z-direction). This size is considered large enough to avoid any inter-slab interactions. In addition, the bottom C-layer of SiC was terminated with H atoms and was held fixed (with its H atoms) during a complete structural relaxation of the rest of the model system. The purpose of these constraints was to simulate a continuation into bulk SiC. During the relaxation process, force minimization took place until the force on each atom was less than 0.005 eV/Å.

The Au adlayer on the Si face of 4H-SiC (0001), and beneath the buffer C layer, was thereafter formed (as shown in Fig. 1). It was either built as a flat monolayer (Fig. 1c) or slightly tilted (within the supercell) to the underlying Si surface (Fig. 1d). Moreover, the Au layer was also mixed with Ag atoms (with 67% Au atoms, and 33% Ag atoms) in the adlayer (Fig. 1e), or built as a monoatomic layer step in the supercell (Fig. 1f).

## Supplementary information

Dataset information.
