# Supplementary material for: The morphology of an intercalated Au layer with its effect on the Dirac point of graphene
Source: Sci Rep. 2020 Jan 23;10:1042. doi: 10.1038/s41598-020-57982-z (PMC6978371; doi:10.1038/s41598-020-57982-z)
Supplement: Supplementary file 1 — Dataset information. [file 41598_2020_57982_MOESM1_ESM.pdf]

Supporting Information

The morphology of an intercalated Au layer with its effect on the Dirac point of graphene

Amirhossein Bayani, Karin Larsson*

Department of Chemistry-Ångström laboratory, Uppsala University, Uppsala, Sweden

**amirhossein.bayani@kemi.uu.se*

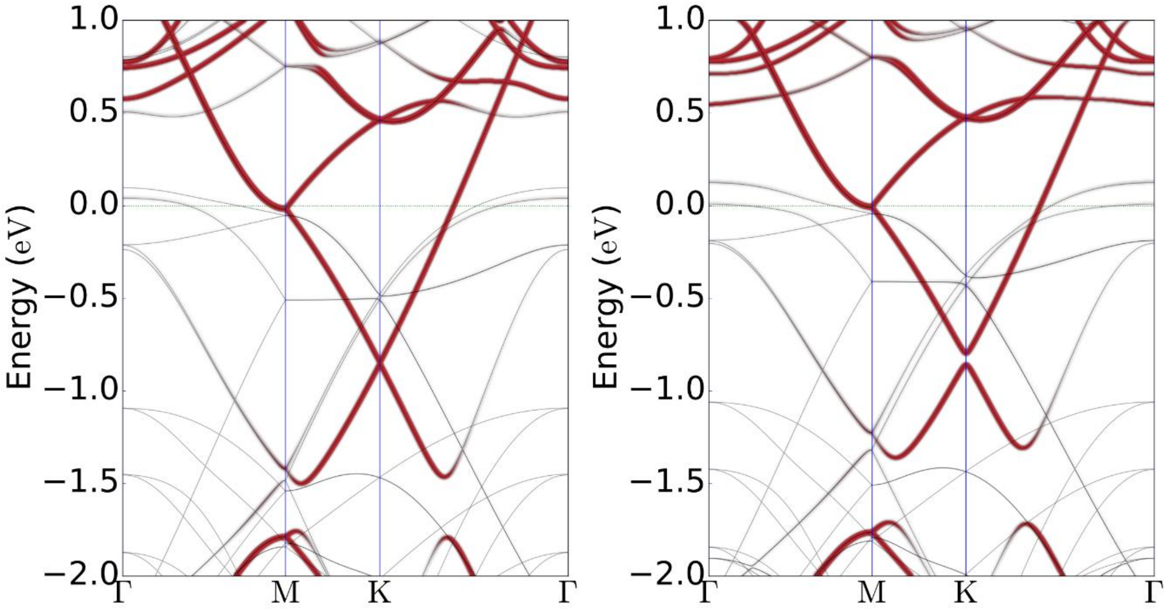

Fig. S1: Projected band structures for large unit cells (shown in Fig. 1b) of flat (a) and tilted Au (with Δ = 0.35 Å) (b) layers within a SiC/Au/graphene supercell structure. The projected band structures for the p_z orbitals of graphene are shown in red, and these band structures have been calculated without spin-orbit interactions. The results show that the asymmetry breakage of the A and B sublattice symmetry is the only important cause of bandgap opening at the Dirac point of graphene. The change in unit cell size will not alter this conclusion.

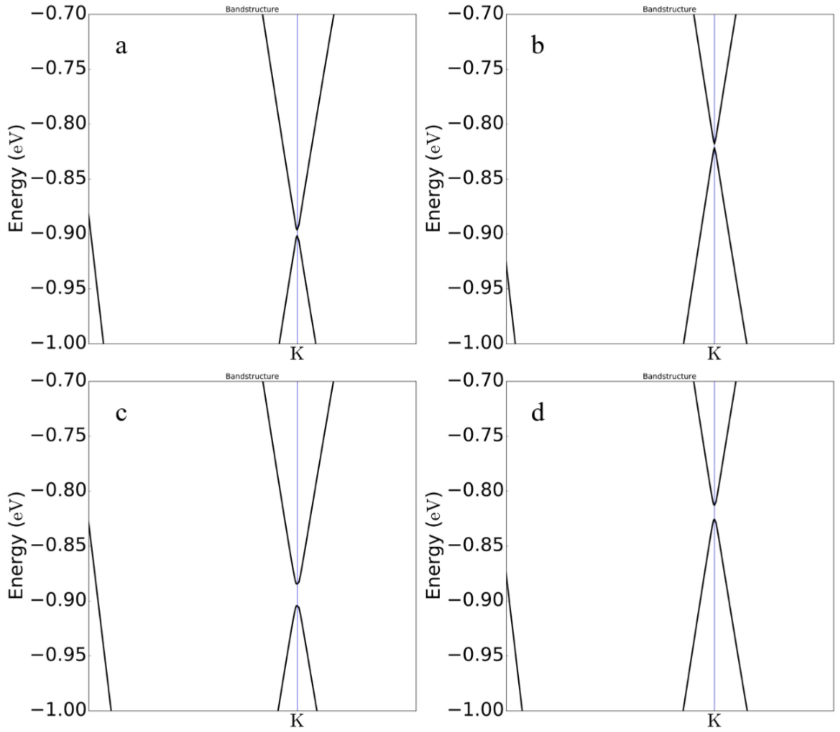

Fig. S2: Band structures show the bandgap opening at the Dirac point of graphene for two different gold-graphene distances: (a),(b) flat Au layers; (c), (d) tilted Au layers with a tilting size (*Δ*) of 0.11 Å within the supercell. (a) and (c) show bandgap openings for a distance of 0.3 nm, and (b) and (d) show a bandgap opening for a distance of 0.32 nm. The bandgap opening at the Dirac point of graphene is obviously induced by an inhomogeneous potential. However, by increasing the distance between the Au layer and graphene, the corresponding bandgaps will decrease. The bandgap sizes presented in (c) and (d) are 19.7 and 12.6 meV, respectively. These values are smaller than the value presented in the main text as a result from the present investigation (30.2 meV).

Another result that is worth mentioning here is that by increasing the gold-graphene distance, the position of the Dirac point will change. (b) shows that the Dirac point of graphene is located at -0.82 eV, while it is located at -0.96 eV for a distance of 0.287 nm (see Table 1 in the main text).

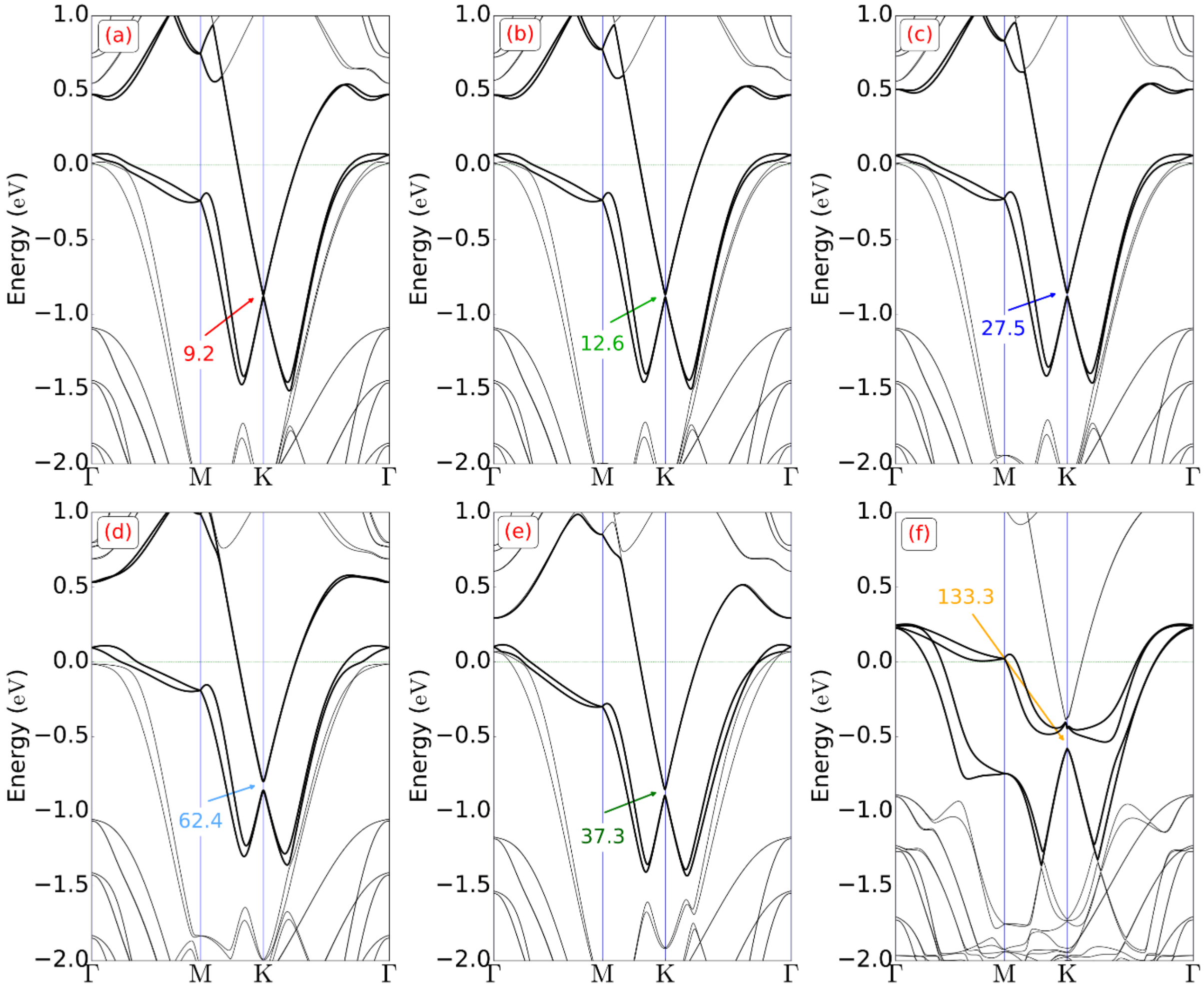

Fig. S3: These bandstructures were obtained when the distance between the Au layer and graphene was set to 3.2 Å. Compared to Fig.2, the values of the energy gap at DP were found to decrease significantly. These results points at a strong relation between the size of the energy gap at DP and the Au-graphene distance. The given values are in meV.

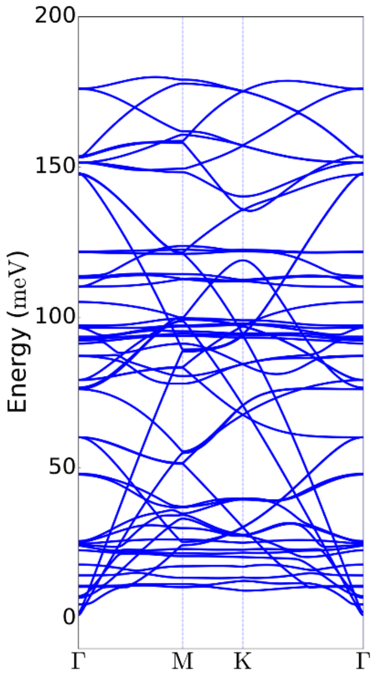

Fig. S4: Phonon bandstructure of SiC/Au/graphene for a zig-zag-shaped Au layer in the Γ-M-K-Γ direction. All phonon branches are positive, with no appearance of imaginary phonon modes. The absence of negative bands proves the stability of the present structure.

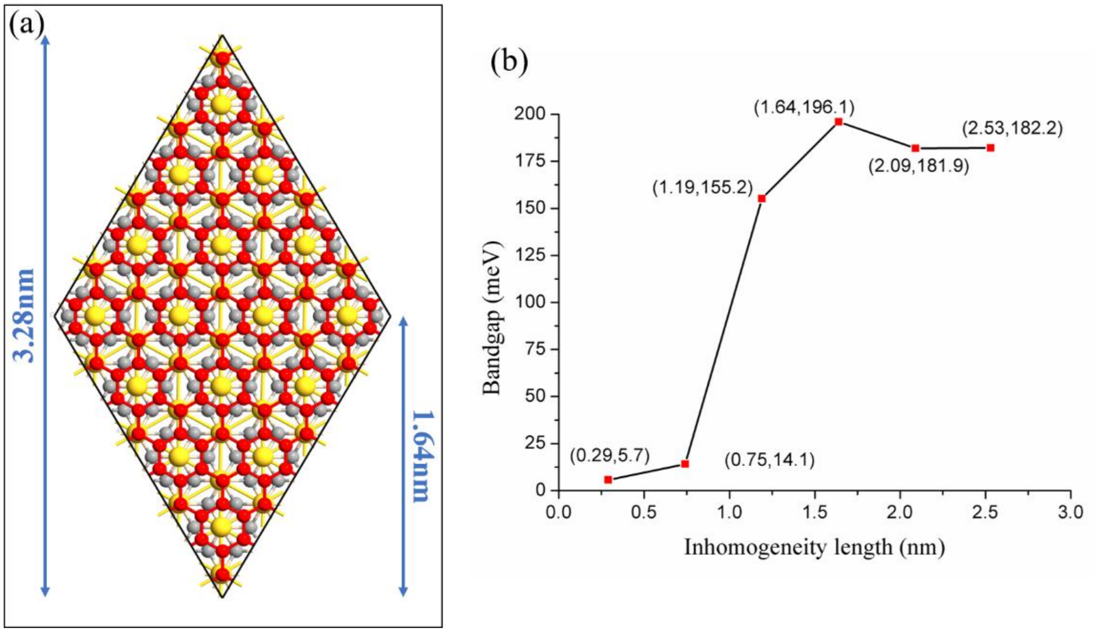

Fig. S5: (a) presentation of the $4\sqrt{3} \times 4\sqrt{3}R30^{\circ}$ unit cell of SiC/Au/graphene which has been used to study the effect of the inhomogeneity length (IL) on the bandgap size at the Dirac point of graphene. In this structure, a (8×8) graphene sheet has been used to match the underneath positioned SiC (in SiC/Au) structure. This new supercell was thereafter used in the construction of periodic 2D models, by successively filling the supercell (from the bottom and upwards) and thereby forming so called inhomogeneity lengths (ILs). ILs were formed from 0.29 nm to 2.53 nm. (b) shows the values for the bandgap (at the Dirac point of graphene) with increasing ILs. The largest bandgap (196.1 meV) was obtained for an IL value of 1.64 nm. This length corresponds to the situation where 50% of the Au sheet is covered with two-layer formations of triangular islands.
